# Video Surveillance Processing Algorithms utilizing Artificial Intelligent (AI) for Unmanned Autonomous Vehicles (UAVs)

**DOI:** 10.1016/j.mex.2021.101472

**Published:** 2021-07-27

**Authors:** Minh T. Nguyen, Linh H. Truong, Trang T.H. Le

**Affiliations:** a Thai Nguyen University of Technology, Viet Nam; b National Tsing Hua University, Taiwan

**Keywords:** Artificial Intelligence, Background Modeling, Region of Interest (RoI), Convolution Neural Networks (CNN), Video Surveillance (VS), Unmanned aerial vehicles (UAVs)

## Abstract

With the advancement of science and technology, the combination of the unmanned aerial vehicle (UAV) and camera surveillance systems (CSS) is currently a promising solution for practical applications related to security and surveillance operations. However, one of the biggest risks and challenges for the UAV-CSS is analysis, process, and transmission data, especially, the limitations of computational capacity, storage and overloading the transmission bandwidth. Regard to conventional methods, almost the data collected from UAVs is processed and transmitted that cost huge energy. A certain amount of data is redundant and not necessary to be processed or transmitted. This paper proposes an efficient algorithm to optimize the transmission and reception of data in UAV-CSS systems, based on the platforms of artificial intelligence (AI) for data processing. The algorithm creates an initial background frame and update to the complete background which is sent to server. It splits the region of interest (moving objects) in the scene and then sends only the changes. This supports the CSS to reduce significantly either data storage or data transmission. In addition, the complexity of the systems could be significantly reduced. The main contributions of the algorithm can be listed as follows;

- The developed solution can reduce data transmission significantly.
- The solution can empower smart manufacturing via camera surveillance.
- Simulation results have validated practical viability of this approach.

The experimental method results show that reducing up to 80% of storage capacity and transmission data.

## Graphical Abstract

**Figure:**
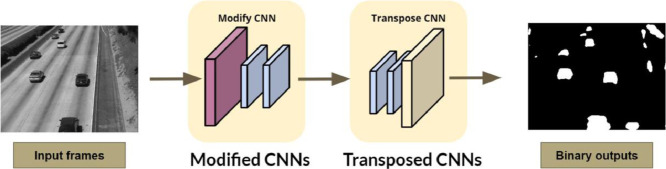

Specification table

**Table:** 

Subject Area	*Computer Science, Data processing, Data communication*
More specific subject area	*Data Surveillance, Object classification and recognition*
Method name	*D-CNN object classification*
Name and reference of original method	Minh T. Nguyen, Linh H. Truong, Trang T. Tran, Chen-Fu Chien, "Artificial intelligence based data processing algorithm for video surveillance to empower industry 3.5", Computers & Industrial Engineering, Volume 148, 2020,106671.
Resource availability	https://doi.org/10.1016/j.cie.2020.106671

## Method details

### Introduction

Over the decades, unmanned aerial vehicles (UAVs) are considered to be an alternative solution, aiming to create the safest working environments to humans from dangerous areas or high-risk missions [1,2]. With the ability to remotely real-time surveillance, UAVs equipped with cameras can capture images or videos to track targets such as people, vehicles or specific areas.

Currently, UAV surveillance has been upgraded with many more features of self-controlling, analysis and data processing by integrating UAVs with artificial intelligence (AI) [3]. Using these technologies, UAVs can be trained to perform particular tasks by processing large amounts of images and videos and simultaneously identifying presence region of interests (RoIs) in frames such as rebar detection bridge deck inspection [4], perform monitoring tasks for early warning of natural disasters [5] or crack detection [6]. Indeed, the integration between UAV with AI technology could help UAV in performing complex tasks rather than surveillance. In addition, AI technology also improve existing limitations of UAV surveillance systems such as storage capacity, processing capability, transmission bandwidth, thus help trasmisting data continuously, reducing computational cost and increase the accuracy of the RoIs detection [7,8].

**Fig. 1:**
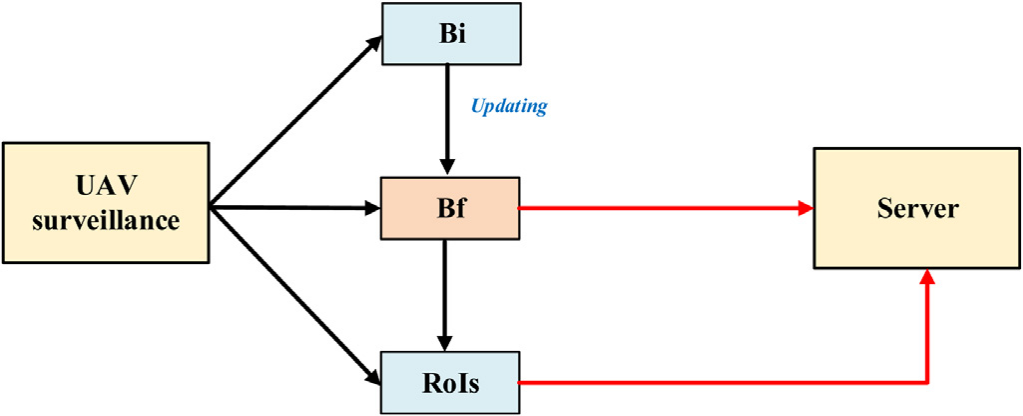
Block diagram

Following to the realistic needs, this study introduces a novel method aiming to distribute more appropriate in the data processing process on both UAVs and Server sides of the UAV surveillance system. The block diagram in Fig. 1 describes the operation steps of the video processing on the UAV surveillance side before sending data to the server-side. Each frame of the input video is used for two purposes, which is performing backgrounds, Bf and region of interest, RoIs. The Bf is referred to the background which is updated if there are static changes over previous frames. In the other hand, ROIs contain moving objects which are obtained by applying background subtraction technique. A special feature is that only the RoIs will be sent to the server. Meanwhile, the background through the updating process from Bi into Bf, the final background. The Bf is then sent to the server-side. Merging Bf and ROI (moving objects) on the server side would generate frames with full background and foreground. Also, this technique applied on the camera side helps the server on the server side only need to focus ROIs to process further tasks. Thereby reducing the computational burden on the server side.

## Data Processing at camera surveillance side

**Fig. 2:**
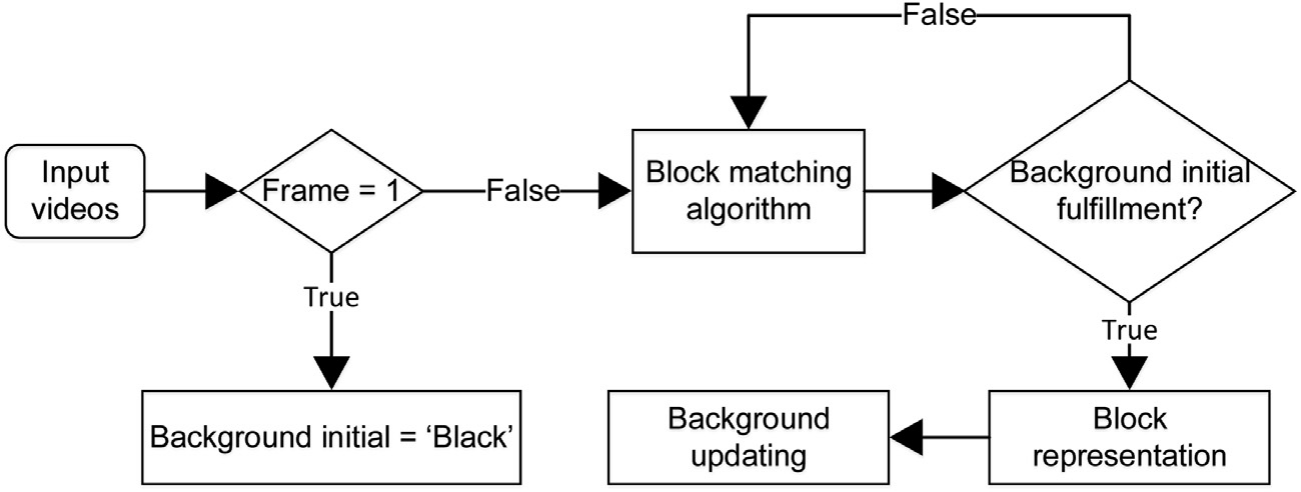
The flowchart of the background initialization

Each frame of original input video with the size of width, *W* and height, *H* has static blocks and might be contained moving blocks. In order to model the background without moving objects, an algorithm is shown in Fig. 2 is proposed. The background is modeled following the t-consecutive video frames, *t* can be varied based on specific scenes. The frames at time t=1 and time t+1 are denoted by $F^{t}$ and $F^{t+1}$, respectively. At the first frame of the input video F^1^ or t = 1, the initial background frame B^t^ is defined as undefined and denotes as “black” blocks. For following frames, the frame t is compared with the previous frame t - 1 by a block matching algorithm to determine moving areas that can be refered as area contained moving objects and create motion maps, $R^{t}$. Block matching algorithm calculates the cost function at each possible location in the search window. This leads to the possible match of the macro-block in the reference frame with a block in another frame. The background, $B^{t}$ are updated based on the previous initial background $B^{t-1}\;$ and motion frames $R^{t}$. The background updating procedure is performed until all undefined blocks are filled by background blocks.

**Fig. 3:**
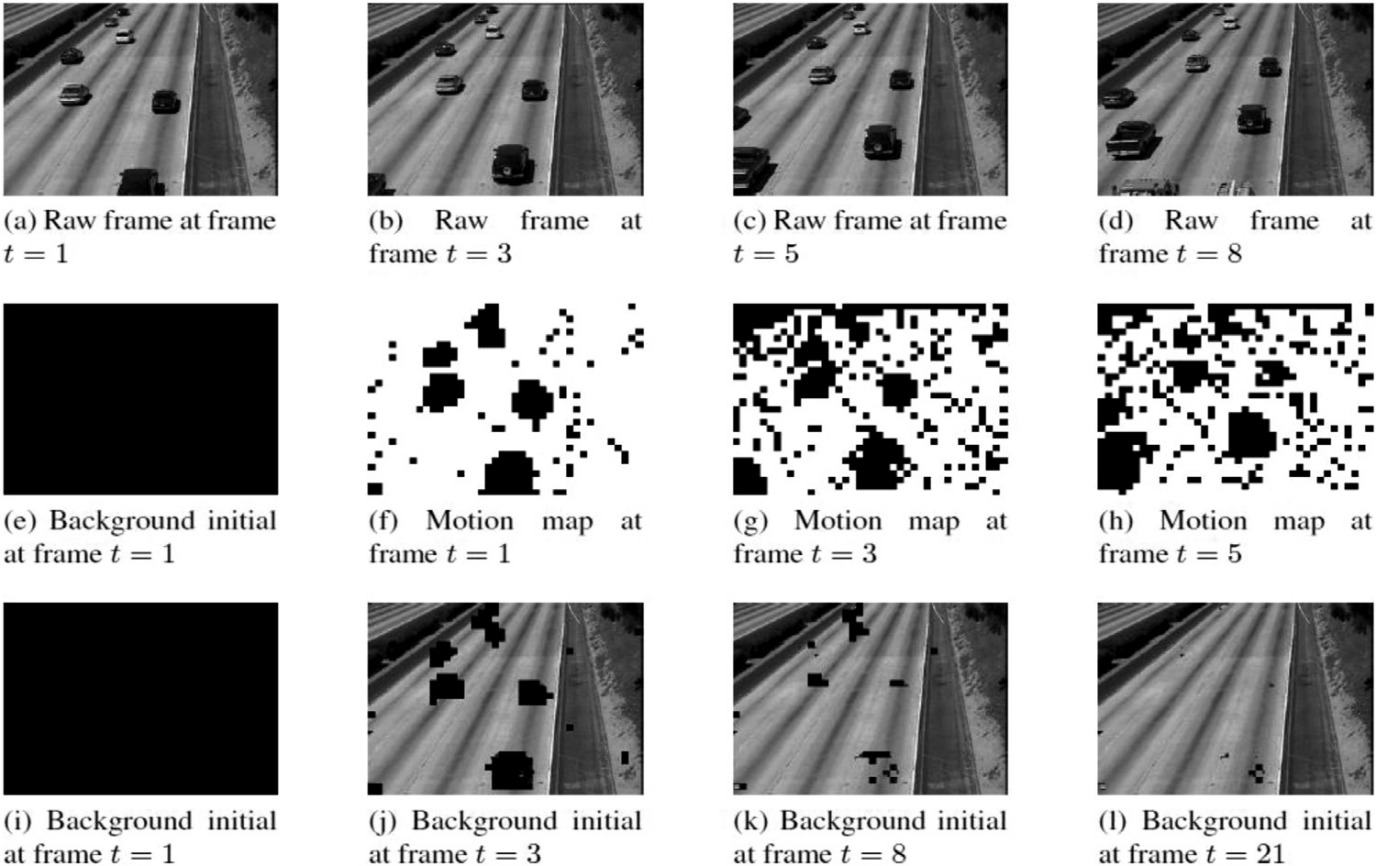
Background initial at first few frames from ATON dataset

As shown in Fig. 3, there are original frames $F^{t}$, motion frame $R^{t}$ in which moving blocks are denoted as “black” blocks and “white” blocks as static blocks, and background initials at different frames. At the first frame, t = 1, the background initial, $B^{1}$ is modeled with all “black” blocks, as shown in Fig. 3e. From t = 2, by applying block matching algorithm, if a frame includes moving objects, the motion map, $R^{t}$ blocks will be marked with “black” blocks, otherwise will be a “white” block. The background modeling process is performed via $R^{t}$ and the previous background, $B^{t-1}$. In addition, at this frame, if block in $R^{t}$ that is “white” block and the corresponding block in the $B^{t-1}$ initial is “black” then those blocks in the background initial will be duplicated from the frame. Similarly, after all “black” blocks in background initial are replace by “white” blocks in $R^{t}$, the background initial is performed. Since then, the background initial will participate in the process of updating the background.

Each block in $F^{t}$could be classified into four different categories as: “background”, “still object”, “environmental change”, and “moving object”. Motion map for each frame, $R^{t}$ exits two states which are background marked in “white” block and movement marked in “black” block. Correlation coefficient *C_B_*, as shown in Eq. 1, is compared with threshold to determine the category of each block in two consecutive frames $F^{t}$, $F^{t-1}$. The block representation of video frame $F^{t}$ is classified as “background” if the block represents of $R^{t}$ is “background”, so if *C_B_ > TH_CB_* or “still object” if *C_B_ < TH_CB_.* Otherwise, for $R^{t}$, if *C_B_ > TH_CB_* the frame block represent is “environmental change”, else is “moving object”.

Correlation coefficient:

(1) $$C_{B(m,n)}=\frac{\sum |b_{m,n}^{t}-\mu_{b_{m,n}^{t}}|\times \left|{\hat{b}}_{m,n}^{t}-\mu_{{\hat{b}}_{m,n}^{t}}\right|}{\sqrt{\sum |b_{m,n}^{t}-\mu_{b_{m,n}^{t}}{|}^{2}}\times \sqrt{\sum |{\hat{b}}_{m,n}^{t}-\mu_{{\hat{b}}_{m,n}^{t}}{|}^{2}}}$$

Background and environment change updating

(2) $${\tilde{b}}_{m,n}^{t}=\alpha {\hat{b}}_{m,n}^{t}+\left(1-\alpha \right)b_{m,n}^{t}$$

Still object updating:

(3) $${\tilde{b}}_{m,n}^{t}=\left\{ \begin{array}{cc} b_{m,n}^{t} & \text{Coun}t_{\left(m,n\right)}\geq TH_{\text{still}}\\ {\hat{b}}_{m,n}^{t} & \text{otherwise} \end{array}\right.$$

Moving object updating:

(4) $${\tilde{b}}_{m,n}^{t}=\left\{ \begin{array}{cc} b_{m,n}^{t} & \text{SM}\left(b_{m,n}^{t}\right)<\text{SM}\left({\hat{b}}_{m,n}^{t}\right)\\ {\hat{b}}_{m,n}^{t} & \text{otherwise} \end{array}\right.$$

Where $b_{m,n}^{t}=\left\{I_{(mN+i,nN+j):i,j=0,1,2,...N-1}^{t}\right\}$ denotes the block index of frame t, $F^{t}$, $b_{m,n}^{t-1}=\left\{F_{(mN+i,nN+j):i,j=0,1,2,...N-1}^{t-1}\right\}$ denote the block index of frame t-1, $F^{t-1}$ and ${\tilde{b}}_{m,n}^{t}=\left\{B_{(mN+i,nN+j):i,j=0,1,2,...N-1}^{t-1}\right\}$ is denote the block index of the background $B^{t}$, ${\hat{b}}_{m,n}^{t}$ denote the block index of the initial background frames, *µ_b_* is the mean of the pixel values in block **b***t*. Additionally $\text{SM}(b_{m,n}^{t})$ and $\text{SM}({\hat{b}}_{m,n}^{t})$ denote the side-match measures for block $b_{m,n}^{t}$ from $F^{t}$ embedded in ${\hat{B}}^{t}$ and that for block ${\hat{b}}_{m,n}^{t}$ embedded in ${\hat{B}}^{t}$, respectively. Note that if a block in ${\hat{R}}^{t}$ is determined as a “moving object” block two or more times continuously.

The next step is extracting moving objects in each frame and transmit to the sever sides. The metrics is applied for the model performance evaluation of ROIs extraction and D-CNN process: F-Measure, is defined as:

(5) $$F-\text{measure}\;=\;\frac{2\times \text{precision}\times \text{recall}}{\text{precision}+\text{recall}}$$

Where $precision=\frac{TP}{TP+FP}$ and $recall\;=\frac{TP}{TP+FN}$. The range of F-measure is between 0 and 1 where if the result of F-measure = 1, that mean the predicted value is totally match the ground truth.

**Fig. 4:**
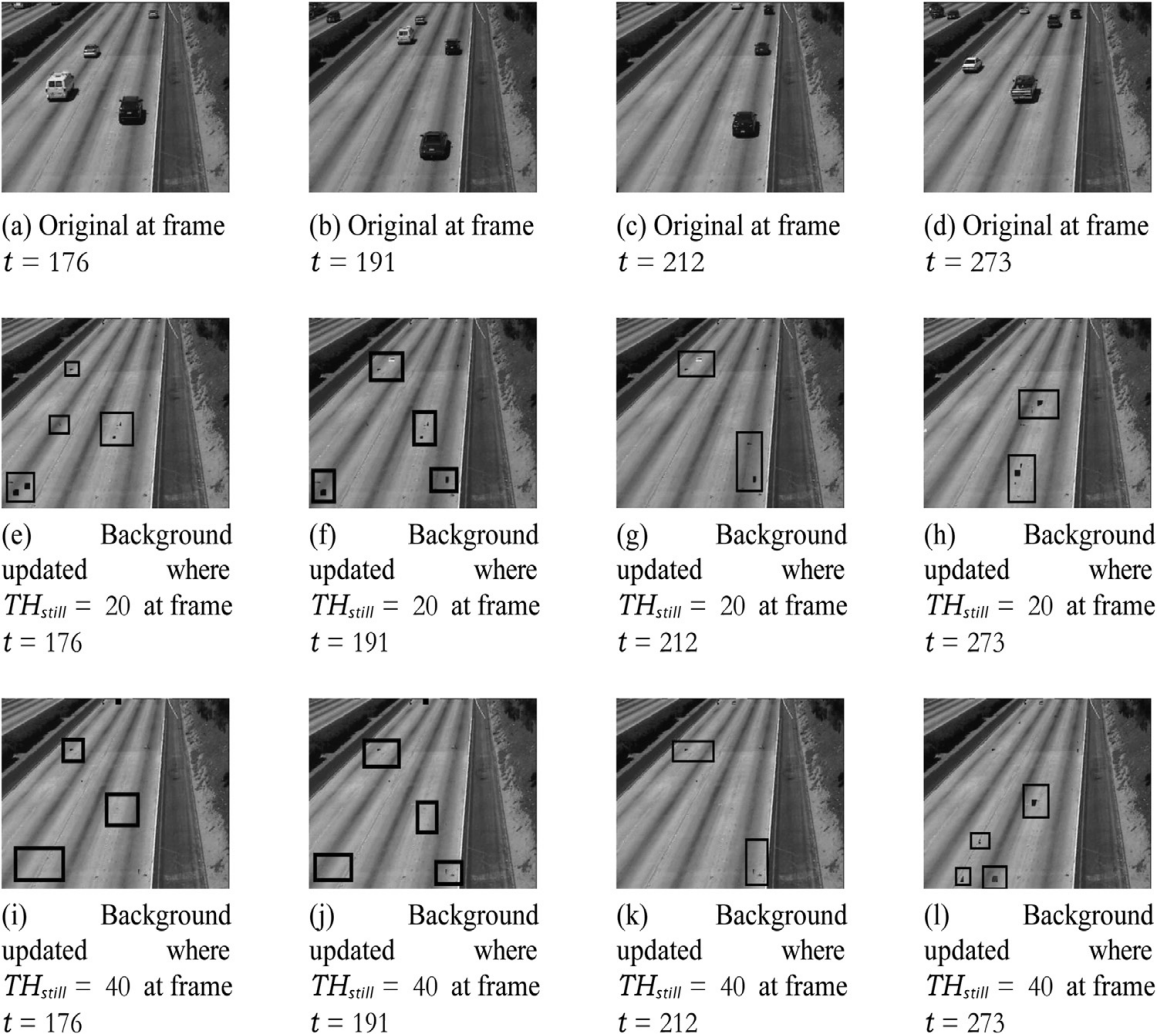
Still object updating for background estimate where a) raw frames t, (b) back- ground updated with TH_still_ = 20 and (c) background updated with TH_still_ = 40 at different times.

The videos used in this paper is taken from the ATON dataset which contains several videos captured from camera surveillance. By using this method, the time to create the background initial is approximate 30 frames, then 170 next frames for background updating that the blocks are “still object” or “illumination change” is updated. Finally, the updated backgrounds will be sent to the server side. Otherwise, it will use the previous background. TH_still_ is a rate to update the background and it can vary for different scenes. The experimental results show that if setting TH_still_ = 20, the background will be updated faster (at frame = 97 after has initialized “background initial”) and vice versa with TH_still_ = 40 (at frame 173), however, the accuracy of TH_still_ = 40 is higher as shown in Fig. 4.

**Fig. 5:**
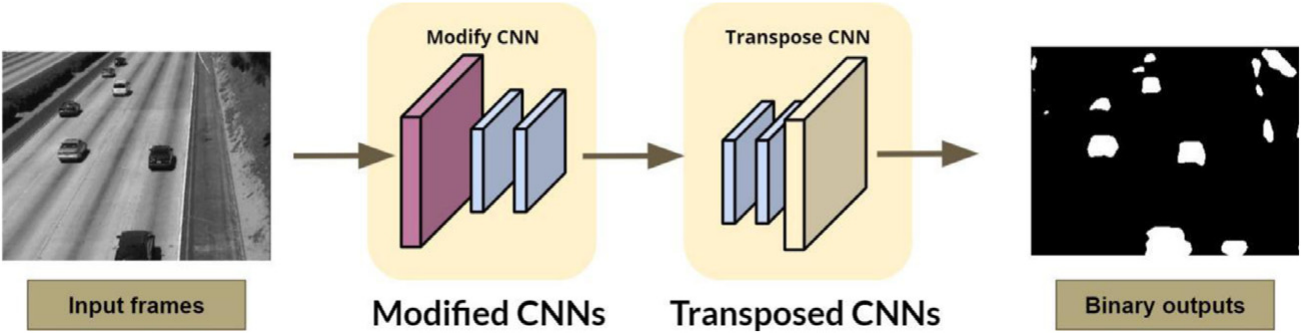
Extracting Region of Interests.

After having background model, the process which is shown in Fig. 5, is separate the foreground from the original frame. Only these foregrounds are sent to the server side. Our network is trained to be able to map raw pixel values from video frames to a set of binary values, between 0 and 1.

Based on the image resolution, it will see W×H×3 (W = Width, H = Height, 3 = RGB color channels). The CNNs models will receive each input frame to train and test. Table 1 depicts the processed steps of network architecture including two stages with many convolutional layers. At the first stage, each frame separated from the video is directly received by a modified CNN network. The 16 feature maps having the kernel size and the stride of 1 at the end of the first convolutional block are transformed from this frame. Next step, the output of the second block is the 32 feature maps of size W/2×H/2. These feature maps are made from the 16 feature maps at the end of the first convolutional block down sampling a max-pooling layer with a stride of 2. The following steps are repeated the same which present in Table 1.

**Table 1:** The Edge DCNN network configuration at the camera side. A modified CNNs (based on U-net network architecture [9]) is from block 1 to 4, where block 0 is RGB input image. In the other side, the edge Transpose-CNN is from block 5 to 9, where block 9 is the output probability mask from the network.

	Edge modified-CNN		Edge Transposed CNN
0	W×H×3, RGB image	10	(Conv) W×H×16,F=3×3,S=1 Final output W×H×1,F=1×1,S=1
1	(Conv) W×H×16,F=3×3,S=1 (Conv) W×H×16,F=3×3,S=1 Max-pooling, F=2×2,S=2 Dropout rate = 0.1		
2	(Conv) $\frac{W}{2}\times \frac{H}{2}\times 32,F=3\times 3,S=1$ (Conv) $\frac{W}{2}\times \frac{H}{2}\times 32,F=3\times 3,S=1$ Max-pooling, F=2×2,S=2 Dropout rate = 0.1	9	(Trans) W×H×16,F=3×3,S=1 Concatenate 9 & 1W×H×32,F=3×3,S=1 Dropout rate = 0.1 (Conv) W×H×16,F=3×3,S=1 (Conv) W×H×16,F=3×3,S=1
3	(Conv) $\frac{W}{4}\times \frac{H}{4}\times 64,F=3\times 3,S=1$ (Conv) $\frac{W}{4}\times \frac{H}{4}\times 64,F=3\times 3,S=1$ Max-pooling, F=2×2,S=2 Dropout rate = 0.1	8	(Trans) $\frac{W}{2}\times \frac{H}{2}\times 32,F=3\times 3,S=1$ Concatenate 8 & 2, $\frac{W}{2}\times \frac{H}{2}\times 64$ Dropout rate = 0.1 (Conv) $\frac{W}{8}\times \frac{H}{8}\times 32,F=3\times 3,S=1$ (Conv) $\frac{W}{8}\times \frac{H}{8}\times 32,F=3\times 3,S=1$
4	(Conv) $\frac{W}{8}\times \frac{H}{8}\times 128,F=3\times 3,S=1$ (Conv) $\frac{W}{8}\times \frac{H}{8}\times 128,F=3\times 3,S=1$ Max-pooling, F=2×2,S=2 Dropout rate = 0.1	7	(Trans) $\frac{W}{4}\times \frac{H}{4}\times 64,F=3\times 3,S=1$ Concatenate 7 & 3, $\frac{W}{4}\times \frac{H}{4}\times 128$ Dropout rate = 0.1 (Conv) $\frac{W}{8}\times \frac{H}{8}\times 64,F=3\times 3,S=1$ (Conv) $\frac{W}{8}\times \frac{H}{8}\times 64,F=3\times 3,S=1$
5	(Conv) $\frac{W}{16}\times \frac{H}{16}\times 256,F=3\times 3,S=1$ (Conv) $\frac{W}{16}\times \frac{H}{16}\times 256,F=3\times 3,S=1$	6	(Trans) $\frac{W}{8}\times \frac{H}{8}\times 128,F=3\times 3,S=1$ Concatenate 6 & 4, $\frac{W}{8}\times \frac{H}{8}\times 256$ Dropout rate = 0.1 (Conv) $\frac{W}{8}\times \frac{H}{8}\times 128,F=3\times 3,S=1$ (Conv) $\frac{W}{8}\times \frac{H}{8}\times 128,F=3\times 3,S=1$

Transposed convolution (TC) uses some learnable parameters to unsampled the input feature map into a desired output feature map. In this case, the input feature map of the TC is the output of the Edge Modified CNN. It is taken to the Transposed CNN (TCNN) to learn the weights for decoding the feature maps. Following the input of TCNN have the size of 16 times smaller than the original size and consists of 256 feature maps with the size of $\frac{W}{16}\times \frac{H}{16}\times 256$. With respect to the block 6 of TCNN stage, the output of modified CNN is projected into $\frac{W}{8}\times \frac{H}{8}\times 128$ by using 2×2 transposed convolution filter with stride of 2.

By applying sigmoid function and threshold the pixel values at the last layer, the output of the transpose CNN is the probability mask for each pixel with the value for noticed objects of 1 and background of 0.

**Fig. 6:**
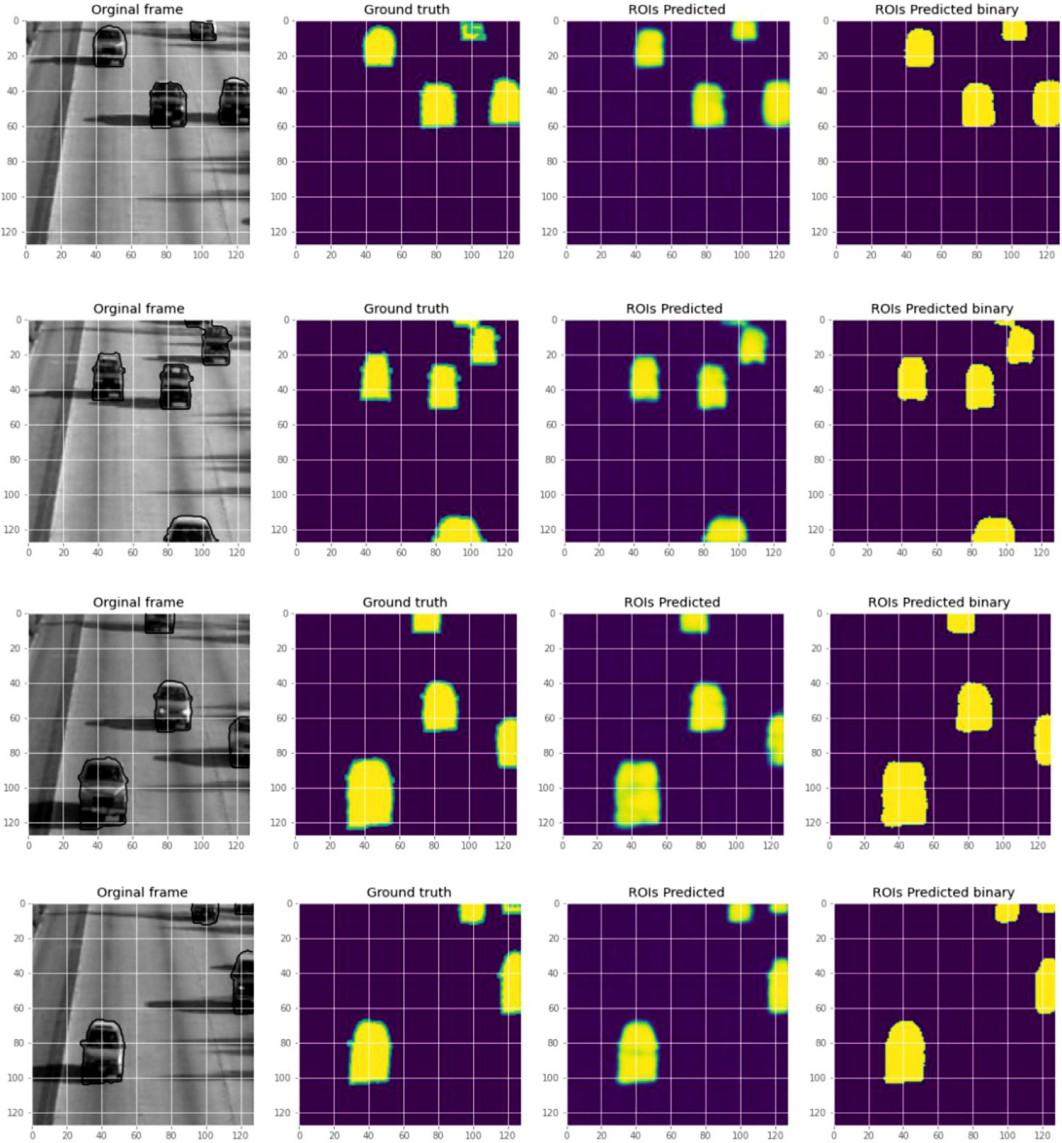
ROIs extraction with findContour at random frames.

The region of interest extraction with findContour is shown in Fig. 6.

**Fig. 7:**
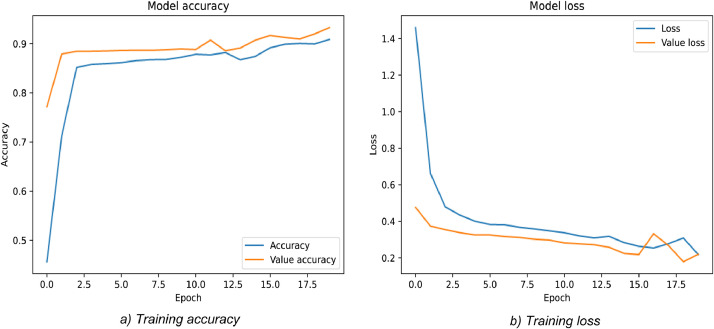
Evaluate training performance at edge side.

About 50 to 200 training examples are employed in this proposed model. From the SBI dataset as training examples, the experiments select random 100 images. There are around 1.2 million parameters in total. The true label compares with the predicted value by using a binary cross-entropy loss function. The RMSProp optimizer with a batch size of 5, 100 epochs are applied to aim with training the network. Based on the description in Fig 7, the model checkpoint helps to improve the network performance and increase the precision of the model. This point does not update until training accuracy increases, or training losses decrease. Hence, this strategy helps the network keep training results better.

## Data Processing at Server Side

**Fig. 8:**
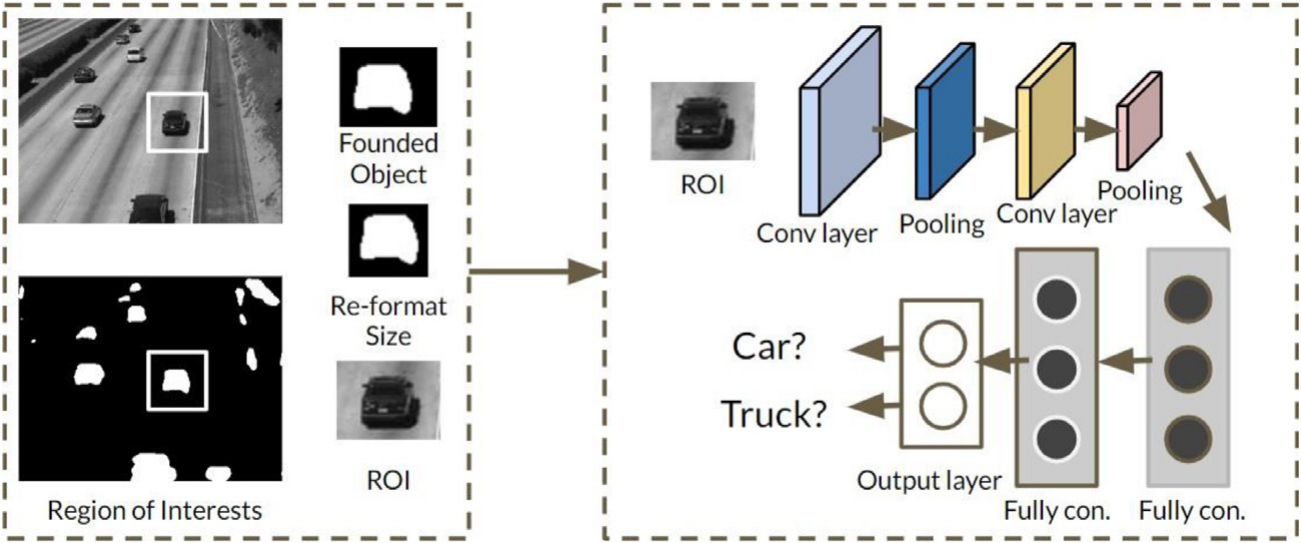
The D-CNN object classification.

The server side receives the RoIs to train their own D-CNN network, as shown in Fig. 8. When the network bases on from those actual data, accuracy and relevance will significantly increase. The first default input size of RoIs is 64 × 64 pixels of RGB to meet the input image requirements from the various ROI size separated from the previous step. In addition, about 200 RoIs is deployed to train DCNN at the server side. This model system needs to go through several experiments to achieve the best performance with the optimal size. When the server receives well-defined RoIs, the system only needs to focus ROIs with the small size and no need for complex classification [11]. All RoIs with the default size selected manually classifies into two classes as “car” and "truck". The classification system consists of two convolution layers, two pooling layers arranged alternately, two fully connected layer and finally the output layer. The rectifier linear units (ReLU) in the final layer plays a role as the active function. The detailed custom classification of the network is given in Table 2.

**Table 2:** The D-CNN network configuration at the sever side.

1	Input layer: 64*64 pixels
2	Convolution layer: W×H×256,F=3×3,S=1 Active function: ReLU Max-pooling layer: $\frac{W}{2}\times \frac{H}{2}\times 256,F=2\times 2,S=1$
3	Convolution layer: $\frac{W}{2}\times \frac{H}{2}\times 256,F=2\times 2,S=2$ Active function: ReLU $\frac{W}{4}\times \frac{H}{4}\times 256,F=2\times 2,S=2$
4	Flatten layer
5	Fully connected layer: Dense 64
6	Fully connected layer: Dense 1
7	Active function: sigmoid

The number of RoIs is divided following two classes 100 ROIs chosen as cars and 100 ROIs as trucks, all extracted from the video in the ATON dataset- 33 seconds of length with 25 frames per second, thus total frames are 825 frames (http://cvrr.ucsd.edu/aton/shad).

**Fig. 9:**
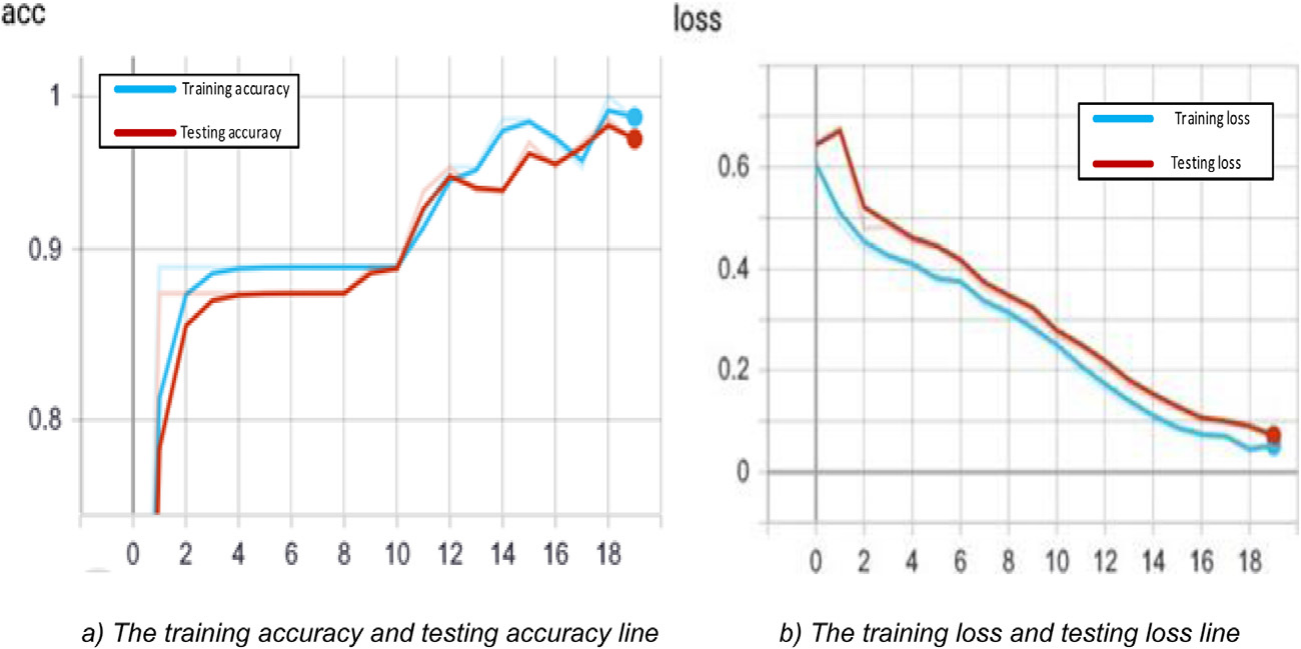
Evaluate training performance of the DCNN at the server side.

To achieve the highest accuracy of the training process, the network need has experimented for several epochs, with an era range from 5 to 20. The best result got an epoch equaling 12 in which the trade-off between training Creating time and accuracy are the same. Fig. 9 shows that at epoch is 12, our DCNN took 13.18 seconds to finish with a loss: 0.0544 - acc: 0.9841 - lost val: 0.1650 - val acc: 0.8571.

In order to calculate the complexity, the number of parameters of each layer has been calculated, that is convolution layer as follow:

(6) $$Number\;of\;parameters\;=\;F^{2}\times C\times K$$

Where F is the filter size, C is the number of input channels, and K is the number of filters. As shown in Table 2, the number of parameters of the custom CNN on the server side can significantly reduce in the network compared to existing object classification and detection methods. At the server edges, the parameters in CNN customized has 209K compared to 66 million or approximately 0.3% of the YOLO method's parameters. In addition, the memory usage per image/frame is 1.7 MB compared to 53.1 MB of the YOLO method.

The classification of the noticed objects bases on evaluating the performance of the custom CNN. In addition, F-measure is also implemented as the measurement. The precision, recall, and F-measure are detailly calculated, as shown in Table 3. With respect to each input image size, the experiment was repeated 10 times using random sub-sampling. Because the size and volume of the data set is smaller, the performance of theis system is not high. In the future, when the amount of data is enhanced, this performance will certainly improve. The measure here is the average of 10 experiments with accuracy, precision and recall. The proposed network shows very good processing results with a smaller number of operations in both training and classification.

**Table 3:** Complexity and memory usage of approaching and a state-of-the-art.

	D-CNN model	YOLO model
*Input size*	64 × 64	224 × 224
*# of conv.layer*	2	24
*# of parameters*	209K	66,000K
*Memory usage/image*	1690KB	53,606KB

**Algorithm 1:** Background updating algorithm.

## Declaration of Competing Interest

The authors declare that they have no known competing financial interests or personal relationships that could have appeared to influence the work reported in this paper.
